# Distracted by the Unthought – Suppression and Reappraisal of Mind Wandering under Stereotype Threat

**DOI:** 10.1371/journal.pone.0122207

**Published:** 2015-03-27

**Authors:** Carolin Schuster, Sarah E. Martiny, Toni Schmader

**Affiliations:** 1 Department of Empirical Educational Research, University of Konstanz, Konstanz, Germany; 2 Department of Psychology, UiT The Arctic University of Norway, Tromsø, Norway; 3 Department of Psychology, University of British Columbia, Vancouver, Canada; Utrecht University, NETHERLANDS

## Abstract

Previous research has found that subtle reminders of negative stereotypes about one’s group can lead individuals to underperform on stereotype-relevant tests (e.g., women in math, ethnic minorities on intelligence tests). This so called stereotype threat effect can contribute to systematic group differences in performance that can obscure the true abilities of certain social groups and thereby sustain social inequalities. In the present study, we examined processes underlying stereotype threat effects on women’s math performance, specifically focusing on the role of suppression of mind wandering (i.e., task-irrelevant thinking) in stereotype threat (ST) and no threat (NT) situations. Based on a process model of stereotype threat effects on performance, we hypothesized that women under stereotype threat spontaneously suppress mind wandering, and that this suppression impairs performance. An alternative regulation strategy that prevents suppression (i.e., reappraising task-irrelevant thoughts as normal) was predicted to prevent stereotype threat effects on performance. We manipulated stereotype threat (ST vs. NT) and cognitive regulation strategy (suppression, reappraisal, or no strategy) and measured women’s performance on a math and a concentration task (*N* = 113). We expected three groups to perform relatively more poorly: Those in ST with either no strategy or suppression and those in NT with a suppression strategy. We tested the performance of these groups against the remaining three groups hypothesized to perform relatively better: those in NT with no strategy or reappraisal and those in ST with reappraisal. The results showed the expected pattern for participants’ math performance, but not for concentration achievement. This pattern suggests that ineffective self-regulation by suppressing mind wandering can at least partly explain stereotype threat effects on performance, whereas a reappraisal strategy can prevent this impairment. We discuss implications for the understanding of processes underlying stereotype threat effects and the benefits of reappraising subjective experience under threat.

## Introduction

“Just focus!” is the advice people are often given before a test. Focusing on a task and shielding one’s mind from distractions is indeed an important skill for academic success [1]. At times, pushing these distractions out of mind is not difficult. If a distraction comes from the outside and requires no action, for example an unfamiliar car alarm goes off in the distance, it can be relatively easy to regain one’s focus by ignoring this interruption [1]. However, test takers can also get distracted by internal states like thoughts unrelated to the task itself [2,3]. For example, they might find themselves thinking about their next meal or about an earlier remark by a friend. These episodes of mind wandering (i.e., being distracted by task-irrelevant thoughts) can result from a failure to maintain attentional focus on a task and lead to superficial processing and reduced cognitive performance [4–6]. Although anyone can experience such distractions, individuals taking a test while being reminded of ways in which they or their group are stereotyped to perform poorly might be especially prone to mind wandering [2]. In fact, past evidence reveals that individuals underperform on stereotype relevant tasks (i.e., women taking a math test) when reminded of those negative stereotypes (a phenomenon known as the *stereotype threat effect*) partially due to mind wandering [2]. At the same time, active efforts to suppress mind wandering might further compromise performance, because suppression relies on domain general executive functions needed to perform well on cognitively demanding tasks [7–9]. Nonetheless, test takers faced with reminders of being negatively stereotyped have been shown to use this costly suppression strategy to cope with internal distractions [10–12].

In this paper, we assert that in contrast to suppression, a more effective strategy is to reappraise episodes of mind wandering and distractions as a normal response during a test. Reappraising mind wandering as a benign experience should discourage suppression efforts that would otherwise impair performance. More precisely, the goal of the current study was to test the hypothesis that not only does suppressing distracting thoughts impair performance (even in the absence of stereotype threat), but that reappraising these thoughts in more benign terms should mitigate the stereotype threat effects on performance. As such, this is the first study to address the performance implications of different regulatory strategies for dealing with episodes of mind wandering. Thus, implications drawn from this research can help test takers perform at their best, especially those disadvantaged by negative performance stereotypes (e.g. women, ethnic minorities).

### Stereotype Threat Effects on Performance

Negative stereotypes exist about many social groups, denying their abilities in certain domains (e.g., “women are bad at math”). Because tests often purport to diagnose one’s ability, they are commonly examined as situations that can pose a threat in the sense that performing poorly might be interpreted as confirming negative stereotypes (i.e., a stereotype threat; e.g., [13]). As research documenting stereotype threat effects has demonstrated, stereotyped group members can perform worse when these stereotypes are brought to mind (e.g., by suggesting that a test is diagnostic of a stereotype-relevant ability) as compared to performance situations that are framed in less stereotype-relevant ways (e.g., [13–15]. Initially, the search for mechanisms underlying stereotype threat effects on performance focused on the negative thoughts and emotions that are directly aroused when people face the possibility of confirming a negative stereotype. Indeed, people report having more negative thoughts about their performance [16,17], self-doubt [13], and anxiety (e.g., [18,19]) when they experience stereotype threat. However, the evidence that the negative content of one’s thoughts and feelings mediates performance impairments due to stereotype threat is mixed at best [20,21].

### Metacognitive Processes Underlying Stereotype Threat Effects

More recent research suggests that the negative thoughts and feelings that are cued under stereotype threat might not themselves undermine performance directly, but rather the effort to detect, interpret, and regulate these experiences is what is particularly debilitating [21]. As such, the present study fits within a larger integrated threat model of stereotype threat [22] by presuming that active meta-cognitive processing and efforts taken to regulate one’s experiences are at least partly responsible for undermining performance in the face of negative stereotypes. Schmader and colleagues [22] argue that the motivation to avoid confirmation of a negative stereotype can lead people to interpret their performance through “threat-colored glasses” ([23], p .16), becoming vigilant for signs of failure and appraising their own reactions in a self-critical manner. Ambiguous outcomes like making an error or feeling somewhat anxious can be easily construed as evidence of failure under stereotype threat. As a result, stereotyped group members become vigilant to detect and then suppress these possible indicators of failure [11,21,24].

### Evidence for Suppression under Stereotype Threat

In line with these ideas, past research has revealed that individuals experiencing stereotype threat try to suppress negative thoughts and emotions related to the experience of stereotype threat [11,12]. These suppression efforts rely on the same working memory resources needed for complex cognitive tasks and thus partly explain why stereotype threat leads to underperformance [22]. Such results are an important practical application of a more general phenomenon whereby thought suppression becomes very difficult under cognitive load (e.g., [9,25]), suggesting that it relies on working memory. Yet, only two papers have examined how suppression processes might undermine performance in situations of stereotype threat. One paper did so by providing indirect evidence of suppression effects to draw inferences about the possible suppression of stereotypes [12], the other focused on suppression of signs of anxiety that would be known by others [11]. No prior research has examined more directly the performance debilitating effects of suppressing unwanted thoughts during a threatening performance situation. Yet this question is perhaps one that more aptly explains people’s subjective meta-cognitive experience at least under subtle induction of stereotype threat. That is, people might find themselves losing focus (rather than having conscious thoughts about stereotypes or even their own anxiety) and try to push out of mind the intrusive and irrelevant thoughts that crop up. If such suppression is spontaneously occurring under stereotype threat, then explicit instructions to push any distracting thoughts out of mind should lead to similar performance impairments among non-threatened participants as we might see among stereotype-threatened participants. Thus, extending basic research on thought suppression and the prior two published papers on emotional and stereotype suppression under stereotype threat, we first hypothesized that efforts to suppress any kind of distracting thought, independent of the activation of a negative performance-related stereotype, would impair performance.

### Reappraisal Prevents Performance Decrements in Threatening Situations

A second aim of the current study was to examine whether reappraisal of mind wandering episodes would be an effective way to alleviate the effect of stereotype threat on performance. Previous research suggests that a more effective way to cope with intrusive and sometimes negative thoughts is to accept rather than suppress them (e.g., [26,27]). More specific to the topic of stereotype threat, earlier research has shown that reappraising anxiety as normal and adaptive (and thus making suppression unnecessary) prevents stereotype threat effects on performance by preventing emotional suppression [11]. Similar strategies work even when they are not targeting specifically negative or stereotype threat-related experiences: thinking about a test objectively instead of as emotionally relevant prevents underperformance under stereotype threat ([11], Study 2), and reappraising arousal improves performance on high stakes quantitative tests (i.e., tests that are potentially threatening even without negative stereotypes; [28]). The effectiveness of reappraising different experiences in different situations suggests that the core difference between performing well or poorly lies in the regulation strategy (*i*.*e*., *suppression or reappraisal*) that individuals use to manage their subjective experience and perhaps not the content of the experience itself (*i*.*e*., *feeling anxious*, *self-doubt*). However, the benefits of reappraising mind wandering under stereotype threat has not previously been examined. In the present research, we thus hypothesized that whereas suppressing mind wandering would impair performance, reappraising mind wandering as a normal occurrence would not. Although it has been argued that mind wandering can be a symptom of executive resource depletion [4], to our knowledge, no work has examined whether the suppression or reappraisal of mind wandering episodes can itself have negative effects on performance.

### The Present Research

In the present study, we experimentally manipulated the cognitive self-regulation strategies (suppression or reappraisal as compared to a third no strategy condition) used by women completing tasks that were either described as diagnostic of math ability (*stereotype threat*, ST) or non-diagnostic (*no threat*, NT). Orthogonal to this standard manipulation of stereotype threat, one third of the sample was instructed to suppress all task-irrelevant thoughts during the tasks (*suppression strategy*), one third were told that it is normal and harmless to have task-irrelevant thoughts during tests (*reappraisal strategy*), and the last third received no strategy information (*no strategy condition*). This design allowed us to address two questions: Firstly, does the suppression of mind wandering impair performance, regardless of the experience of stereotype threat? Because suppression is generally a cognitively demanding regulation strategy [7,8], we expected poor performance from women instructed to suppress their experience of mind wandering even if they were in the no threat (NT) condition. However, we also argue that ST spontaneously triggers people’s motivation to suppress task-irrelevant thoughts [12]. Thus, we expected women under stereotype threat (ST) to perform poorly if given no strategy instruction or if instructed to suppress unwanted thoughts. Thus, we expected similarly poor performance in the ST/no strategy, the ST/suppression, and the NT/suppression groups, compared to the NT/no strategy condition, which should serve as a reference group for optimal performance from the sample (see Table 1).

**Table 1:** Predicted Relative Performance Levels of the Experimental Groups.

	No Strategy	Suppression	Reappraisal
Stereotype Threat	-	-	+
No Threat	+	-	+
Hypotheses	*Stereotype threat undermines performance*	*Suppression undermines performance*	*Reappraisal protects performance*

The experimental groups with a minus sign are expected to perform worse than the groups with a plus sign.

Secondly, can stereotype threat effects on performance be prevented by reappraising mind wandering episodes as benign? In line with previous studies showing that reappraisal is generally beneficial for performance [13,29] and can prevent suppression of the target experience ([11], Study 4), we expected that a reappraisal of task-unrelated thoughts would prevent underperformance in the face of ST, and would lead to better performance than the suppression strategy even in a no threat condition. Thus, we hypothesized that participants in the ST reappraisal, NT reappraisal, and NT no strategy conditions would perform similarly, and better than the thought suppressors (ST no strategy, ST suppression, NT suppression).

We tested these predictions on two different performance measures: one was a difficult math reasoning test with problems adapted from the quantitative section of the Graduate Record Exam (GRE), which has been shown to be sensitive to stereotype threat effects [30]. The other task was a concentration test [31] featuring addition and subtraction problems completed under time pressure and thus highly dependent on working memory. Consequently, performance on this task was expected to be sensitive to stereotype threat [32]. Although these tests require different skills, we expected that self-regulation processes under stereotype threat would affect performance on each in a similar manner.

## Method

### Ethics Statement

The procedures of the experiment were in compliance with the Ethical Principle of the WMA Declaration of Helsinki. No formal approval was required by the Institutional Review Board of the University of Konstanz previous to the experiment, because there was no reason to assume that the procedures could entail any lasting harms or risks for the participants. Students volunteered to participate in an experiment on ‘how to make tests more fair’ for a 6 € reward. Before the experiment started, they signed a written consent form following the ethical code of conduct of the American Psychological Association [33]. The questionnaires and the data files only contained anonymous data. After the experiment, all participants received detailed information about the hypotheses in order to give them the chance to withdraw their consent on that basis, which no one did. In addition, by informing our participants about the nature of stereotype threat effects on performance, we intended to protect them from such effects on future tests [34].

### Participants and Design

One hundred and fifteen female students (*M*_*age*_ = 21.60, *SD* = 2.53) at five universities of applied sciences were randomly assigned to a 2 (stereotype threat: ST vs. NT) by 3 (strategy: suppression vs. reappraisal vs. no strategy) between-subjects design. The dependent variables were performance on a math test and a concentration test. There were no performance differences between affiliates of the five universities on the math test, *F*(108,4) = 1.36, *p* = .25, nor the concentration test, *F* (108,4) = 1.40, *p* = .24. The participating students came from a wide range of majors, many of them in the areas of economics, computer science, healthcare, and their intersections (e.g., Business Informatics, Medical Engineering, Healthcare Management). Two participants were excluded from analyses for failing to follow test instructions. In the final sample, there were 19 participants in each condition, except for the ST-suppression group (n = 18).

### Procedure

As part of an ostensible study of test design, participants learned that they would complete two diagnostic math intelligence tests (Stereotype Threat) or non-diagnostic math practice tests (No Threat; e.g., [13]). For each test they would have eight minutes. Participants who were in the suppression or reappraisal conditions received additional information as part of the instructions for Test 1, telling them that thoughts unrelated to the task might come up and they should try to suppress them (suppression condition) or that such thoughts are normal and harmless (reappraisal condition). The strategy manipulation was repeated briefly on the instruction for Test 2. Participants in the no strategy condition received no instructions concerning distracting thoughts. An English translation of the exact wording of the manipulation is available as supporting information (S1 Manipulations). Following the two tests (which were counterbalanced), participants completed a final questionnaire, were thoroughly debriefed, and rewarded with 6 € and chocolate.

### Measures

#### Math performance test

The math test contained eight comparison problems where participants have to decide which of two values derived from text or an equation is greater [30]. A pilot study (*N* = 34) showed that the problems were perceived as difficult (*M* = 5.06, *SD* = 0.89, on a 6-point scale; percentage solved correctly ranged from 8.8% to 60.8%). As in the Graduate Record Exam (GRE), the final performance score was the number of correct answers adjusted for guessing; that is, a fourth of the number of wrong answers was subtracted from the number of correct answers, such that test scores could range from -2 (i.e., 8 wrong answers) to 8 (i.e., 8 correct answers).

#### Concentration Achievement Test

A shortened version of the Concentration Achievement Test [31] was included as a second performance measure. In this speeded test, participants have to mentally calculate two terms (e.g., 8–2+3 and 4+9–5). If the first interim result is larger, they have to subtract the second interim result from the first one (9–8 = 1). If the second interim result is larger, they have to add the two interim results. As all operations have to be processed without writing interim results down, this is a task highly dependent on working memory. In total, the test contained 80 problems split into 4 columns with a time limit of two minutes for each. Participants solved on average 26.01 (*SD* = 9.46) problems and 20.34 of them correctly (*SD* = 9.60). We used the number of correct answers as the dependent variable.

#### Domain identification

Because high domain identification has been shown to be a necessary pre-condition for stereotype threat [16,35], we included a math identification measure. Participants answered four math identification items on a Likert-Scale ranging from *not at all true* (1) to *absolutely true* (6) (e.g., *It is important for me to be good in math*., α = .81, [36], p.40). Participants’ average response was significantly above the scale midpoint (*M* = 4.25, *SD* = 0.92), *t*(111) = 8.64, *p* < .01, *d* = 0.79, suggesting that, on average, participants in this sample should be susceptible to experiencing stereotype threat.

#### Perceived difficulty of performance tests

High difficulty has also been shown to be a pre-condition for stereotype threat [35], thus we asked participants to rate the difficulty of each test on a 6-point Likert-Scale (*The first/second test was difficult for me*). Perceived difficulty of the math test was significantly above the scale midpoint (*M* = 4.84, *SD* = 1.02), *t*(110) = 17.47, *p* < .01, and was higher than the perceived difficulty of the concentration test (*M* = 3.12, *SD* = 1.21), *t*(111) = 12.83, *p* < .01, *d* = 1.00, which was rated less difficult then the scale midpoint, *t*(119) = -2.89, *p* < .01. Thus, the pre-condition of high difficulty might only be given for the math test, but not for the concentration test.

## Results

**Fig 1:**
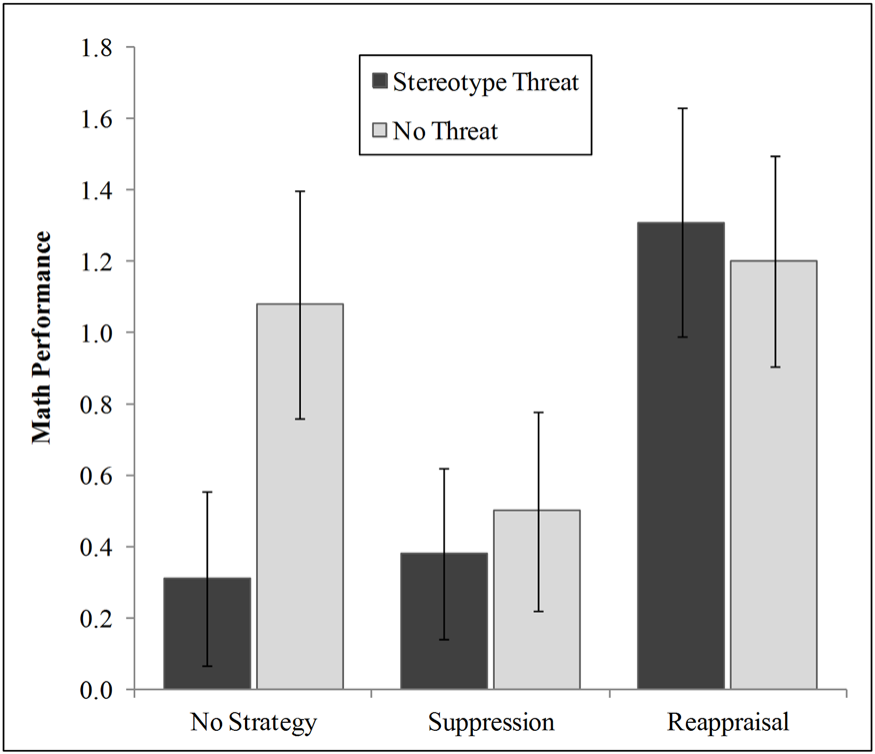
Group Means and Standard Deviations of Math Test Performance. *N* = 113. Numbers at the bottom of the columns are the coefficients of the significant focal contrast. The non-significant orthogonal contrasts were (0 -1 0 1 0 0), (-1 0 0 0 2 -1), (-1 0 0 0 0 1), and (0 -1 2 -1 0 0). The means and standard deviations are, from left to right: *M*_*1*_ = 0.29, *SD*_*1*_ = 1.01, *M*_*2*_ = 1.08, *SD*_*2*_ = 1.40, *M*_*3*_ = 0.38, *SD*_*3*_ = 1.02, *M*_*4*_ = 0.50, *SD*_*4*_ = 1.22, *M*_*5*_ = 1.29, *SD*_*5*_ = 1.40, *M*_*6*_ = 1.20, *SD*_*6*_ = 1.29.

Our hypotheses assume that the variability of performance in our sample represents two distributions: those who perform relatively poorly because they suppress thoughts (either due to stereotype threat or because they were directly instructed to suppress thoughts), and those who perform relatively better because they do not suppress thoughts (either because they are in a no threat condition or because they were instructed to reappraise negative thoughts). Thus, as can be seen in Table 1, we expected three of the six groups to perform poorly because they suppress thoughts: the no-strategy/diagnostic group and both suppression groups (diagnostic and non-diagnostic). The other three groups were expected to perform comparatively better because they do not suppress thoughts: the no-strategy/non-diagnostic group and both reappraisal groups (ST and NT). To increase power, we calculated planned contrast analyses that assigned contrast weights to each condition in way that represents our hypothesized group differences (1 –1 –1 –1 1 1). Specifically, a contrast weight of ‘-1’ was assigned to the groups we expected to underperform, and ‘+1’ was assigned to the groups we expected to perform relatively better. Thus, the contrast analysis tested if the three experimental groups with the same contrast weight stem from a different distribution than the ones with the other coefficient. As suggested by Abelson and Prentice [37], four (*df*-1) sets of orthogonal contrasts (see Fig. 1) were tested for significance in order to rule out systematic variance independent of the proposed focal contrast; thus, we expected the focal contrast to be significant and the orthogonal contrasts to be non-significant (for the same procedure see [38]).

### Math Performance

The contrast analyses with math performance as a dependent variable supported our hypothesis (see Fig. 1): The focal contrast was significant, *t*(107) = 3.45, *p* < .01, and of a moderate size, r_EffectSize_ = .32, CI [.14; .47]. The effect size correlation is the correlation of the contrast weights with the participants’ scores [39]. Consequently, the contrast explains 10% of variance in math scores (*r^2^*_effect size_ = .10). All the orthogonal contrasts were non-significant, all *t*s(107) < .59, *p*s >.56. The effect size correlations of the orthogonal contrasts were *r*_1_ = .02, CI [-.16; .20]; *r*_2_ = .00, [-.18; .19]; *r*_3_ = .05 [-.14; .23], *r*_4_ = .05 [-.14; .23]. The group means in Fig. 1 illustrate, first, a pattern of means consistent with a typical stereotype threat effect on performance among participants in the no strategy condition (i.e., poorer performance of the ST than the NT group when no strategy was given), *t*(36) = 2.00, *p* = .05. Second, they show that thought suppression led to similarly low performance, whether or not stereotype threat was elicited, *t*(35) = 0.34, *p* = .74. And third, the reappraisal strategy, which makes suppression unnecessary, eliminated the stereotype threat effect on math performance, *t*(36) = -0.21, *p* = .83.

### Concentration Achievement

Contrast analyses did not support the hypothesis for performance on the concentration task (see Table 2). The focal contrast was not significant, *t*(107) = -0.52, *p* = .60, neither were the orthogonal contrasts, *t*s (107) = [-0.48; 0.83], *p*s = [.41; .85]. The estimated means and standard deviations for all conditions are summarized in Table 2. Analyses of number of completed answers and accuracy, which are further criteria for performance on this test [31], led to similar results: in all cases the focal contrast and the orthogonal contrasts were not significant, all *p*s ≥ .20.

**Table 2:** Group Means and Standard Deviations of Number of Correct Answers in the Concentration Achievement Test (N = 113).

	No Strategy		Suppression		Reappraisal	
	*M*	*SD*	*M*	*SD*	*M*	*SD*
Stereotype Threat	19.68	7.74	22.33	18.62	21.32	12.07
No Threat	19.32	10.00	20.47	10.67	19.00	8.67

### Control Variables

We ran several additional analyses to control for unintentional influences on these results. First, we examined domain identification as a covariate of the analyses of math and concentration performance. This did not affect the pattern of results: The focal contrast on math performance was still significant, *F*(105,1) = 12.19, *p* < .01, whereas all the orthogonal contrast are non-significant, *p*s > .54. For the concentration test, none of the contrasts was significant, *p*s > .38. Domain identification was not correlated with math performance, *r* = .13, *p* = .17, or number of correct answers on the concentration test, *r* = .09, *p* = .33, and there were no differences in domain identification between groups, *F*(108,5) = 0.44, *p* = .82. Second, we analyzed whether test order had an effect on performance using an omnibus ANOVA with ST, strategy, and order as factors. Order had no significant main or interactive effects on performance on neither the math test, nor the concentration test, all *p*s > .10. Finally, including the two non-compliant participants in the sample had no effect on the results. The focal contrast on math performance was still significant, *t*(109) = 3.39, *p* < .01, whereas all the orthogonal contrast are non-significant, *p*s > .56. Also, there are no significant effects of the focal and orthogonal contrasts on concentration achievement, *p*s > .48.

## Discussion

Generally speaking, the present research examined the effects of suppression and reappraisal of mind wandering on women’s performance under conditions designed to induce stereotype threat or not. The expected performance debilitating effects of stereotype threat and suppression on women’s performance were found on a difficult math test. The results support the hypothesis that those participants who suppressed their episodes of mind wandering performed more poorly than those who did not. The differentiation between non-suppressors (NT no strategy, ST reappraisal, NT reappraisal) and suppressors (ST no strategy, ST suppression, NT suppression) explained 9% of variance with a confidence interval ranging from 2% to 22%. Looking at the patterns of individual group means more closely, we found that among those given no explicit strategy instruction, women in the stereotype threat condition tended to perform worse than in the no threat condition. This pattern is consistent with other work showing the performance-debilitating effects of stereotype threat [14]. One explanation for this effect is that women under stereotype threat might spontaneously suppress internal distractions [22]. Consistent with this account, women under stereotype threat performed similarly poorly regardless of whether they were instructed to suppress mind wandering. Thus, consistent with past work [11,12], suppression as a regulation strategy was found to be detrimental to performance. Here we examined suppression not of stereotypes or anxiety, but of task-irrelevant thoughts that need not be directly about one’s performance.

The other novel contribution from this research is evidence that the reappraisal of mind wandering is a regulation strategy that is beneficial to performance, even when experiencing stereotype threat. Specifically, women told to reappraise irrelevant thoughts as a normal aspect of test-taking performed better than those instructed to suppress regardless of stereotype threat. Those in the stereotype threat condition who were instructed to reappraise mind wandering as benign also performed just as well as women in the no threat/no strategy condition, suggesting that reappraisal of mind wandering alleviated the performance debilitating effects of stereotype threat. Together these patterns of mean performance across condition lend further support to the idea that the stereotype threat impairs performance by cuing cognitively demanding suppression processes, but also shows this ineffective regulation strategy can be beneficially substituted with a reappraisal of the distracting thoughts.

### Limitations of the Present Study

Of the two performance tests we administered, only the math test showed the predicted pattern of performance. On the concentration test, we did not find evidence of a stereotype threat effect, nor the predicted effects of suppression and reappraisal on performance. It has to be noted that this measure was rated by the participants as relatively easy. Previous research has shown that stereotype threat effects on performance appear only on difficult tests that raise uncertainty about one’s capability [13,35]. Similarly, suppressing mind wandering can only be expected to have a negative effect on performance when one is highly cognitively invested in the test. In other words, the moderate difficulty of the concentration achievement test might have made the information given in the manipulations seem less relevant to this task. Tentatively, we might suggest that the effects found on the math test would not generalize to tests which are perceived to be easy, although future work is needed to test this idea directly.

Another limitation of this experiment is that we did not include direct measures of mind wandering or suppression, and thus cannot be certain how the strategy manipulations affected the content or process of participants’ thinking. The conclusions we draw are based on inferences drawn from the effects of the manipulations on math performance. Although these inferences are grounded in theoretical models of stereotype threat [22] and suppression [9] processes, and on previous research on the cognitive costs and benefits of suppression and reappraisal of anxiety [11], future research could complement these results with direct measures of the regulation strategy used. In a similar vein, future work could employ thought listing paradigms to ascertain the degree to which suppression and reappraisal of distracting thoughts harms and helps performance, respectively, by affecting the degree to which task-irrelevant thoughts (e.g., thoughts about other aspects of one’s day) and/or meta-cognitive thoughts (e.g., “I am bad at this task”) distract from the more critical task-relevant thoughts (e.g., “3 times 14 is 42”) essential for successful performance.

### Implications for Understanding Stereotype Threat Processes

The findings of the present work are important in two ways. First, they support the hypothesis that stereotype threat leads to underperformance at least partly due to the suppression of mind wandering. Previous research has shown that people spontaneously suppress stereotype-related thoughts [12] and expressed anxiety [11] under stereotype threat. In addition, suppression of thoughts is an effortful cognitive strategy which competes with other tasks for executive resources (e.g., [9]). Extending and integrating these findings, we showed that suppression of mind wandering is similarly harmful to threatened and not threatened participants’ performance and found a performance pattern that suggests that only participants under stereotype threat use a suppression strategy spontaneously. Such evidence advances our understanding of stereotype threat effects on performance by suggesting that the process of self-regulation is crucial for the typically observed underperformance, whereas the content of distracting thoughts might be less directly associated to performance impairments than previously suggested [13,16]. Even individuals who find their thoughts wandering off to random things like their lunch or a past conversation when reminded of being negatively stereotyped [2], instead of specifically worrying about their ability, will thus underperform if they also attempt to suppress these thoughts. Our findings suggest further that people who suppress mind wandering due to other reasons than stereotype threat, for example because they generally believe they need to control their thoughts [26], will have difficulty performing up to their potential. Interestingly, previous research has devoted little attention to the side effects of thought suppression on performance (only one paper examined memory performance: [8]), but rather has focused on its ineffectiveness in reducing intrusive thoughts [25,40, 7]. The present findings thus emphasize the importance of adequate self-regulation of thoughts. Further research is needed to determine the generalizability of the suppression effect on performance across test situations.

Second, we found that reappraising distracting thoughts prevents participants under stereotype threat from underperforming. Our findings thereby complement previous research showing the effectiveness of reappraising negative experiences such as anxiety [11] or social adversity [29] as normal. Compared to other interventions against stereotype-threat-related underperformance (e.g., teaching about stereotype threat effects [34], retraining attitudes [41], or giving a self-affirming task [42]) the reappraisal intervention can be applied very easily in testing situations. Besides reducing the motivation to suppress [11], reappraising one’s thoughts and emotions as normal might also benefit negatively stereotyped participants in other respects. For example, it might reduce stress [26], mitigate the experienced level of threat, or, like a training to mindfully accept all experiences, actually reduce mind wandering itself [3]. In addition, reappraisal of arousal has been shown to generally benefit individuals’ performance on a high stakes math test [28]. This is in line with the argument made in the introduction that processes affecting performance under stereotype threat (i.e., suppression and reappraisal) can likely be applied to a broader range of performance situations where an important goal is at stake. Steele [43], for example, mentioned the similarity of processes underlying stereotype threat effects and effects of evaluation apprehension, test anxiety, or choking under pressure, on performance. As the effect of suppression and reappraisal seems to be independent of stereotype threat-related content, reappraisal of internal distractions might help in these situations as well [44]. Further research is needed to examine whether reappraisal benefits performance across these circumstances, in order to develop a reliable theory on reappraisal interventions that can be used by practitioners and test takers.

In summary, the present research supports the integrated process model of stereotype threat effects on performance [22]. Specifically, it corroborates the hypothesis that the spontaneously applied strategy to suppress distracting thoughts leads to underperformance under stereotype threat, because it is generally a bad strategy for highly demanding tests. Stereotype threat effects can be prevented by regulating distracting thoughts more effectively. For test takers this implies that in order to perform well, it might be more effective to accept task-irrelevant thoughts as normal and harmless instead of suppressing them. This might not be relevant in all cognitive tests, but seems to apply beyond stereotype threat contexts.

## Supporting Information

S1 Manipulations Experimental manipulations of stereotype threat and strategy. This word document contains the test instructions for all conditions, translated to English.
(DOC)

S1 Dataset Data and documentation of data. This zipped folder file contains the SPSS dataset (S1a_Dataset.sav) of the experiment and an explanatory document (S1b_DatasetDocumentation.txt).
(ZIP)
