# Supplementary material for: Distracted by the Unthought – Suppression and Reappraisal of Mind Wandering under Stereotype Threat
Source: PLoS One. 2015 Mar 27;10(3):e0122207. doi: 10.1371/journal.pone.0122207 (PMC4376717; doi:10.1371/journal.pone.0122207)
Supplement: S1 Manipulations — This word document contains the test instructions for all conditions, translated to English. (DOC) [file pone.0122207.s001.doc]

STEREOTYPE THREAT CONDITIONS

**Mathematical Intelligence Tests**

Now two tests follow, which are scientifically proved to measure natural mathematical abilities. The tests have different problem types; however, they both can assess mathematical intelligence reliably.

This way, your strengths and weaknesses in the mathematical domain can be assessed accurately, and can be compared to those of other men and women with a similar language processing speed.

Please try to be at you best performance.

On the next page you will find an example problem for Test 1.

**Mathematic Intelligence Test 1**

**Example**

In the first line of the problem consists of general information.

Then two columns with the values A and B follow. You need to compare these two values. In every problem choose the correct answer out of four possibilities describing the result of this comparison.

These possible answers are the same in all problems.

| A number of n bowlers have an average score of 160. The average of these n points together with a score of 170 is 161. | |
| --- | --- |
| A: n | B: 10 |
| 1. The quantity on the left (A) is greater 2. The quantity on the right (B) is greater 3. Both are equal 4. The relationship cannot be determined without further information | |

Solution:

Average of n points = sum of all points / number of points

161 = (160 x n + 170) : (n+1) | x (n+1)

160 n + 170 = 161 n + 161 | -161 -160n n = 9 < 10

The correct answer is b)

On the following pages you will find 8 problems of this kind.

You have 8 minutes. Try to solve as many problems as possible in that time. The goal is to be both fast and accurately.

*(no strategy condition: no further information)*

*(or suppression condition:)*

While you are working on the problems thoughts may occur to you that are not directly related to solving the problem but concern other things.

Please try to suppress such irrelevant thoughts as well as possible.

*(or reappraisal condition:)*

While you are working on the problems thoughts may occur to you that are not directly related to solving the problem but concern other things.

Such thoughts are normal in test situations and have no negative effect on the result.

Please do not turn over yet! Wait for signal to start!

**Mathematic Intelligence Test 2**

The second test also is a scientifically proven method of assessing mathematical intelligence.

The following test paper contains arithmetical problems. The following two examples show how to solve them.

| Example A: 8 - 3 + 7 First calculate the upper term and remember the result (= 12).  5 + 6 - 3 Proceed likewise with the lower term (= 8).  Keep both numbers in mind! |
| --- |

1. Rule: Is the upper number greater (as in example A) then subtract the lower one from the upper one (12 - 8) and write the result in the rectangle (= 4).

| Example B: 8 + 5 - 7 Again calculate the upper term and remember the result (= 6).  4 + 9 - 5 Proceed likewise with the lower term (= 8).  Keep both numbers in mind! |
| --- |

2. Rule: Is the upper number less (as in example B) then add the lower one to the upper one
(6 + 8) and write the result in the rectangle (= 14).

So you need to keep both interim results in mind and either subtract or add them!

You must not write down the interim results on the worksheet!

Now calculate calmly the sample problems.

5 - 2 + 6 9 + 5 + 8 9 + 8 - 6 3 + 6 - 5

4 + 7 + 5 6 - 3 + 4 5 - 3 + 5 5 + 7 - 4

On the following pages you find 4 columns with each 20 arithmetical problems of this kind.

Proceed in each column from the top to the bottom. Start with the first column on the left. After two minutes the experimenter will give a signal; stop immediately with the current column and continue to calculate the problems in the next one. After another two minutes, you will be given another signal to start promptly with the next column. Try to work as fast and accurately as you can.

*(no strategy condition: no further information)*

*(or suppression condition:)*

Please try again to **suppress** **irrelevant thoughts** as good as possible.

*(or reappraisal condition:)*

Thoughts that are unrelated to the problem solving are **normal** in this test as well and **do not have any negative effect** on the results.

**Please do not turn over yet! Wait for signal to start!**

NO THREAT CONDITIONS

**Mathematical Practice Tests**

Now two tests follow, which are used in this study as examples for mathematical problem solving. The tests have different problem types, which have not been examined in detail yet.

This way, the strengths and weaknesses of different problem types can be examined for the first time, and can be compared with regard how well they are suited for men and women with different language processing speeds.

Please try to be at you best performance.

On the next page you will find an example problem for Test 1.

**Mathematic Practice Test 1**

**Example**

In the first line of the problem consists of general information.

Then two columns with the values **A and B** follow. You need to **compare these two values.** In every problem choose the correct answer out of four possibilities describing the result of this comparison.

These **possible answers** are the **same in all problems.**

| A number of n bowlers have an average score of 160. The average of these n points together with a score of 170 is 161. | |
| --- | --- |
| A: n | B: 10 |
| 1. The quantity on the left (A) is greater 2. The quantity on the right (B) is greater 3. Both are equal 4. The relationship cannot be determined without further information | |

**Solution:**

Average of n points = sum of all points / number of points

161 = (160 x n + 170) : (n+1) | x (n+1)

160 n + 170 = 161 n + 161 | -161 -160n n = 9 < 10

The correct answer is **b)**

On the following pages you will find 8 problems of this kind.

You have 8 minutes. Try to solve as many problems as possible in that time. The goal is to be both fast and accurately.

*(no strategy condition: no further information)*

*(or suppression condition:)*

While you are working on the problems thoughts may occur to you that are not directly related to solving the problem but concern other things.

Please try to **suppress** such **irrelevant thoughts** as well as possible.

*(or reappraisal condition:)*

While you are working on the problems thoughts may occur to you that are not directly related to solving the problem but concern other things.

Such thoughts are **normal** in test situations and have **no negative effect** on the result.

**Please do not turn over yet! Wait for signal to start!**

**Mathematic Practice Test 2**

The second test also is not yet scientifically examined but a form of problem-solving that is to be investigated here.

The following test paper contains arithmetical problems. The following two examples show how to solve them.

| Example A: 8 - 3 + 7 First calculate the upper term and remember the result (= 12).  5 + 6 - 3 Proceed likewise with the lower term (= 8).  Keep both numbers in mind! |
| --- |

1. Rule: Is the upper number greater (as in example A) then subtract the lower one from the upper one (12 - 8) and write the result in the rectangle (= 4).

| Example B: 8 + 5 - 7 Again calculate the upper term and remember the result (= 6).  4 + 9 - 5 Proceed likewise with the lower term (= 8).  Keep both numbers in mind! |
| --- |

2. Rule: Is the upper number less (as in example B) then add the lower one to the upper one
(6 + 8) and write the result in the rectangle (= 14).

So you need to keep both interim results in mind and either subtract or add them!

You must not write down the interim results on the worksheet!

Now calculate calmly the sample problems.

5 - 2 + 6 9 + 5 + 8 9 + 8 - 6 3 + 6 - 5

4 + 7 + 5 6 - 3 + 4 5 - 3 + 5 5 + 7 - 4

On the following pages you find 4 columns with each 20 arithmetical problems of this kind.

Proceed in each column from the top to the bottom. Start with the first column on the left. After two minutes the experimenter will give a signal; stop immediately with the current column and continue to calculate the problems in the next one. After another two minutes, you will be given another signal to start promptly with the next column. Try to work as fast and accurately as you can.

*(no strategy condition: no further information)*

*(or suppression condition:)*

Please try again to **suppress** **irrelevant thoughts** as good as possible.

*(or reappraisal condition:)*

Thoughts that are unrelated to the problem solving are **normal** in this test as well and **do not have any negative effect** on the results.

**Please do not turn over yet! Wait for signal to start!**
